# Cervical Dysgenesis: A Rare Mullerian Duct Anomaly

**DOI:** 10.7759/cureus.18279

**Published:** 2021-09-25

**Authors:** Subha R Samantaray, Ipsita Mohapatra

**Affiliations:** 1 Obstetrics and Gynecology, Prathima Institute of Medical Science, Karimnagar, IND; 2 Obstetrics and Gynecology, All India Institute of Medical Sciences Kalyani, Kalyani, IND

**Keywords:** congenital anomaly, coring and drilling technique, cervicovaginal canalization, vaginal agenesis, cervical dysgenesis

## Abstract

Cervical agenesis or dysgenesis is a rare congenital anomaly. The patients usually present with primary amenorrhoea, primary infertility, and cyclical abdominal pain or with a history of prior surgeries like hymenectomy, vaginoplasty, or adhesiolysis for endometriosis along with well developed secondary sexual characters. We present a case of 15 years old girl with cervical dysgenesis and proximal vaginal agenesis, who presented with severe cyclical abdominal pain. She was managed with cervicovaginal canalization by coring and drilling technique done by vaginal approach with simultaneous laparoscopic guidance. Being a rare type of developmental anomaly of the female genital tract, no standard treatment for type-2 cervical dysgenesis has been established. The patient was followed up for 18 months during which she reported to be having regular menstruation.

## Introduction

Cervical agenesis or dysgenesis is a rare congenital anomaly with an incidence of about one in 80,000-100,000 live births [1]. The patients usually present with primary amenorrhoea, primary infertility, and cyclical abdominal pain or with a history of prior surgeries like hymenectomy, vaginoplasty, or adhesiolysis for endometriosis along with well developed secondary sexual characters [2,3].

Here we present a case of 15 years old girl with cervical dysgenesis and proximal vaginal agenesis, who presented to our institute with severe abdominal pain. She was managed with cervicovaginal canalization by coring and drilling technique done by vaginal approach with simultaneous laparoscopic guidance. The patient was followed up till eighteen months during which the patient reported to be having regular menstrual cycles.

## Case presentation

**Figure 1:**
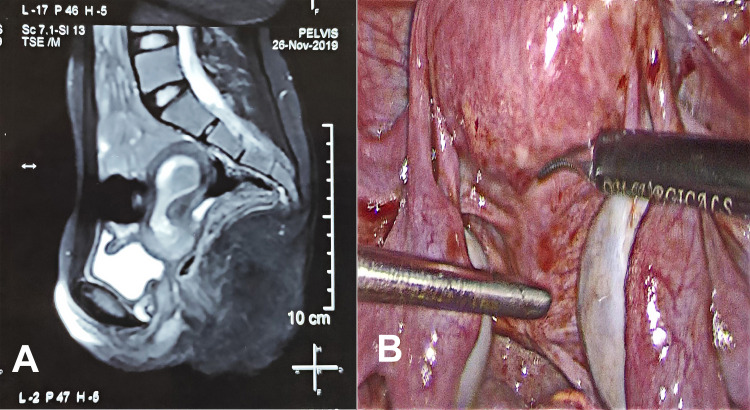
(A) MRI showing the sagittal section of the pelvis showing hematometra, cervical dysgenesis, and atresia of the proximal part of the vagina. (B) Posterior view of the cervix with focal areas of endometriosis.

A 15-year-old female was referred to our institute with severe lower abdominal pain. The patient had a history of cyclical abdominal pain and primary amenorrhea. Her height was 145 cm, weight 41 kg and she had normal secondary sexual characters. Examination under local anesthesia revealed a blind-ending vagina of about 5-6 cm in length and the cervix was not visible through the vagina. Investigations revealed normal routine blood tests, hormonal profiles, and karyotype 46XX. Ultrasonography demonstrated a retroverted uterus with hematometra extending up to the upper part of the cervix; non-canalized lower part of the cervix, absence of proximal part of the vagina, and normal adnexa. These findings were corroborated by magnetic resonance imaging (MRI) of the pelvis which demonstrated a normal endometrial cavity with hematometra extending into the upper part of the cervix (Figure 1A). The lower part of the cervix was fibrotic without any endocervical canal, absent external os and the absence of a proximal portion of the vagina. There was no communication between the cervix and vagina. A diagnosis of cervical dysgenesis with proximal vaginal atresia was made. The urological system was found to be normal.

The patient and her attendees were explained about the condition. After proper counseling and written informed consent, the patient was prepared for surgery. Under general anesthesia, the patient was placed in a lithotomy position at an angle of 30° at the hips. Abdominal entry was done with one 10 mm primary port at the umbilicus and four secondary 5 mm ports (two lateral ports on the left side, one lateral port on the right side, and one suprapubic port). On inspection, minimal altered colored blood collection and few endometriotic spots were noted in the pelvis. Bilateral ovaries and other abdominal viscera were normal in appearance. On the posterior aspect, the uterus with cervix was appearing normal in size and shape along with normal ligamentous attachments (Figure 1B).

**Figure 2:**
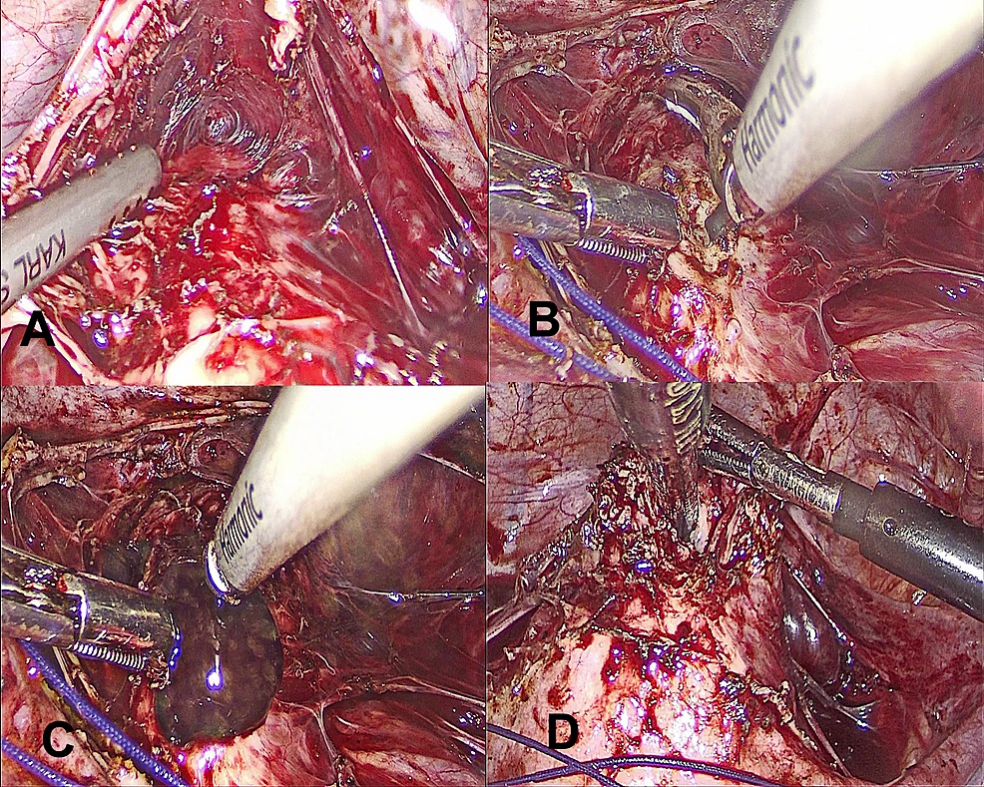
(A) Dissection of bladder away from the cervix. (B) Incision over the cervix. (C) Escape of dark-colored blood through the incision. (D) Maryland grasper negotiated through the cervical incision to dissect the bridging tissue.

The bladder was dissected away from the uterus in the midline (Figure 2A). The upper part of the cervix was incised at midline with the help of a harmonic scalpel (Figure 2B). Dark-colored blood present in the endometrial and endocervical cavity was cleared out (Figure 2C).

Through the suprapubic port, a Maryland grasper was inserted into the cervical canal to identify the lowermost end of the cervical canal and the level of obstruction. The bridging tissue between the cervix and blind vagina was delineated and dissected partly by finger dissection through the vaginal route and partly by a Maryland grasper from above (Figure 2D), to create a neovagina.

**Figure 3:**
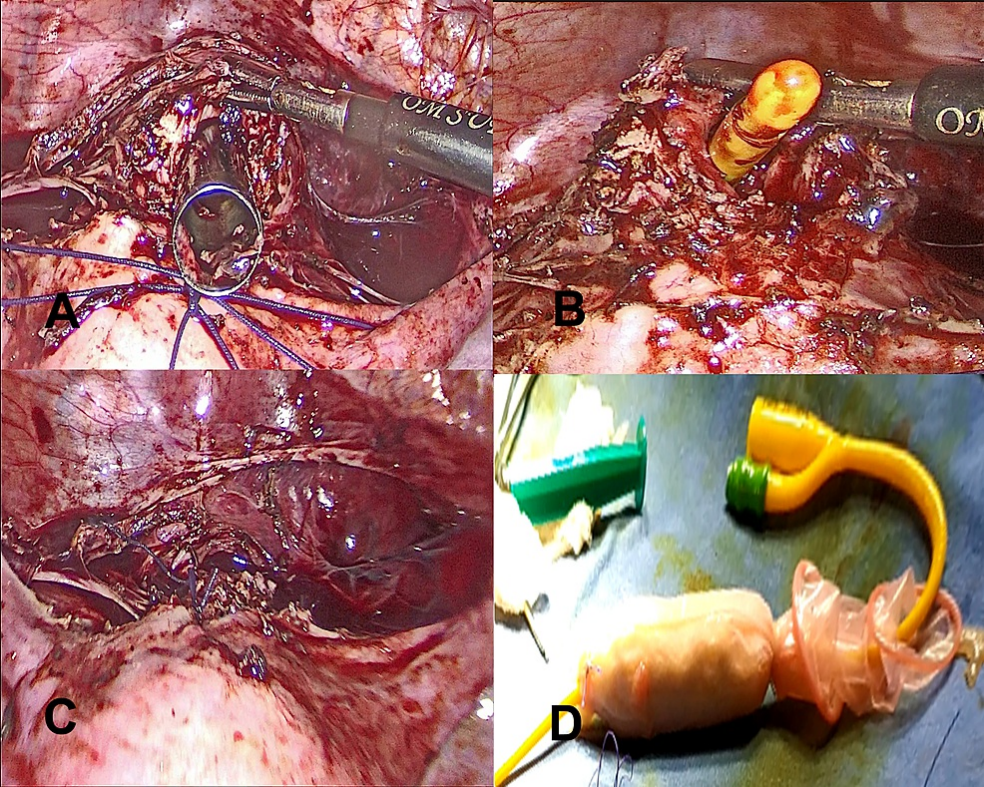
(A) 10 mm trocar visible through the cervical incision. (B) Foley’s catheter was inserted into the newly formed tract. (C) Closure of the cervical incision. (D) Vaginal mold.

The Maryland grasper was negotiated through the remaining thin bridging tissue to make a small channel through it. The Maryland grasper was further advanced under finger guidance till it was visible through the vaginal opening. The outer sheath of a 10 mm trocar was loaded over Maryland and advanced gently through the vaginal route to dilate the passage (Figure 3A). Consequently, a connection between the upper part of the cervix and vagina was established.

A self-fabricated vaginal mold was made over a 5 mL syringe with 14 French Foley catheter at the center and wrapped with sterile gauge pieces and a condom (Figure 3D). This mold was covered with a two-layer amnion graft harvested and prepared from another seronegative patient, who had been delivered by elective cesarean section at the same time. Amnion graft was used for the facilitation of quick and better epithelization of the newly formed tract. This mold was put through the neovagina with the tip of Foleys in the endometrial cavity and dilated with 30 mL of distilled water (Figure 3B). The cervical incision was closed with polyglactin 910 no 1-0 suture in a tension-free manner (Figure 3C). The catheter was left in place to maintain the patency of the newly created tract.

The post-operative period was uneventful. Vaginal mold was removed on the eighth post-operative day. The patient was discharged on the ninth post-operative day. The catheter was kept in situ for four weeks. Till 18 months of follow-up, the patient reported to be having normal menstruation and on examination, the patency of the newly created track was well maintained.

## Discussion

Cervical agenesis or dysgenesis is a rare congenital disorder and limited literature is available about this condition. There is a lack of uniformity in the classification of these kinds of congenital disorders. The complete formation and development of the female reproductive tract have three phases of development; organogenesis, fusion, and septal resorption [4]. Our case had defective septal resorption due to which there was non-canalization of a part of the cervical region and upper part of the vagina resulting in distal cervical dysgenesis and proximal vaginal atresia. According to the new ESHRE/ESGE classification, cervical anomalies can be classified into five classes: C0 (normal cervix), C1 (septate cervix), C2 (double normal cervix), C3 (unilateral cervical aplasia), and C4 (cervical aplasia) [5]. Rock et al. classified cervical anomalies into two basic categories [6]. Patients having type 1-cervical aplasia; lacks a uterine cervix. Type 2-cervical dysgenesis is divided into subtypes: (a) Cervical body consisting of a fibrous band of variable length and diameter. (b) Intact cervical body with obstruction of the cervical os. (c) Stricture of the mid-portion of the cervix with a bulbous tip and no identifiable cervical lumen. (d) Fragmentation of the cervix. Therefore according to this classification, our case has type 2(b) cervical dysgenesis with agenesis of the proximal part of the vagina. Cervical agenesis is commonly associated with vaginal atresia and renal tract anomalies [7].

In the case of obstructive uterine anomalies, the normal menstrual flow is occluded resulting in primary amenorrhea as seen in our case. Patients may be asymptomatic or may present with symptoms like hematometra, hematocolpos, and cyclical lower abdominal pain. Sometimes due to retrograde menstrual flow, the patients may present with signs and symptoms of endometriosis, chocolate cyst, and acute emergencies of ovarian torsion [3].

Diagnosis of cervical dysgenesis with proximal vaginal agenesis was made in our case based on history, clinical findings and further confirmed by ultrasonography and MRI. Ultrasonography is usually the first performed diagnostic modality for Mullerian developmental defects, however, MRI is considered as the gold standard [8]. Other conditions considered as differential diagnoses are imperforated hymen and transverse vaginal septum [5].

The treatment and surgical corrective methods are divisive. It depends on the presenting symptoms, type of anomaly, and the level of obstruction. For type-2 cervical dysgenesis, three types of surgical techniques have been described [9]. They are coring and drilling technique (CDT), uterovaginal anastomosis (UVA), and hysterectomy. A systematic review of 19 articles with a total of 68 patients with cervical dysgenesis, reported that the majority were managed with CDT (55%), 32% underwent UVA and only 13 % were treated with hysterectomy [10]. Conservative surgeries like CDT and UVA can be attempted where a part or the whole cervix with mucosa is present. The aim of this surgery is the restoration of normal anatomical integrity, preservation of functional cervical canal with a theoretical advantage of protection from ascending infection, and preservation of fertility. The overall success rate of conservative surgery is 60% and few cases are reported to have pregnancy; however, it is also known to be associated with various complications like endometritis, pelvic inflammatory disease, persistent pelvic pain, bowel and bladder injury, re-obstruction, and stenosis requiring reoperation and death [9,11]. But a solid conclusion cannot be drawn from these observations. The present case was managed by cervicovaginal canalization with coring and drilling techniques. The connection between the cervix and vagina was done by vaginal approach and simultaneous laparoscopic guidance. As the case was also associated with proximal vaginal agenesis, an amnion graft was used to enhance the epithelization of the proximal neovagina. A Foley catheter was kept for four weeks in the uterine cavity to maintain the patency of the channel made surgically. The index case was successfully managed with conservative surgery and patency was maintained till 18 months of follow-up. Hysterectomy may be preferred as an initial management option for patients with cervical agenesis with fragmentation [6,11]. It may also be done in cases that have failed or complications with any of the conservative methods [12,13].

## Conclusions

Being a rare type of developmental anomaly of the female genital tract, no standard treatment for type-2 cervical dysgenesis has been established. Most of the patients present with primary amenorrhea and cyclical abdominal pain. The management of this condition should be individualized keeping in mind the symptoms and the level of obstruction. Proper counseling of the patient and her family about the condition, possible surgical outcomes, and future fertility issues should be discussed before any intervention is planned.
